# Classical Swine Fever Virus N^pro^ Antagonizes IRF3 To Prevent Interferon-Independent TLR3- and RIG-I-Mediated Apoptosis

**DOI:** 10.1128/JVI.01136-20

**Published:** 2021-02-10

**Authors:** Samuel Hardy, Ben Jackson, Stephen Goodbourn, Julian Seago

**Affiliations:** a The Pirbright Institute, Pirbright, Woking, Surrey, United Kingdom; b Institute for Infection and Immunity, St. George’s, University of London, London, United Kingdom; Hudson Institute of Medical Research

**Keywords:** IRF3, PRR, apoptosis, host-pathogen interactions, innate immunity, interferons, mitochondria, viral pathogenesis

## Abstract

Classical swine fever virus (CSFV) is the causative agent of classical swine fever, a notifiable disease of economic importance that causes severe leukopenia, fever, and hemorrhagic disease in domesticated pigs and wild boar across the globe. CSFV has been shown to antagonize the induction of type I interferon (IFN), partly through a function of its N-terminal protease (N^pro^), which binds interferon regulatory factor 3 (IRF3) and targets it for proteasomal degradation. Additionally, N^pro^ has been shown to antagonize apoptosis triggered by the double-stranded RNA (dsRNA) homolog poly(I·C); however, the exact mechanism by which this is achieved has not been fully elucidated. In this study, we confirm the ability of N^pro^ to inhibit dsRNA-mediated apoptosis and show that N^pro^ is also able to antagonize Sendai virus (SeV)-mediated apoptosis in PK-15 cells. Gene-edited PK-15 cell lines were used to show the dsRNA-sensing pathogen recognition receptors (PRRs) TLR3 and RIG-I specifically respond to poly(I·C) and SeV, respectively, subsequently triggering apoptosis through pathways that converge on IRF3 and culminate in the cleavage of caspase-3. Importantly, this IRF3-mediated apoptosis was found to be dependent on transcription-independent functions of IRF3 and also on Bax, a proapoptotic Bcl-2 family protein, through a direct interaction between the two proteins. Deletion of IRF3, stable expression of N^pro^, and infection with wild-type CSFV were found to antagonize the mitochondrial localization of Bax, a key hallmark of the intrinsic, mitochondrial pathway of apoptosis. Together, these findings show that N^pro^’s putative interaction with IRF3 is involved not only in its antagonism of type I IFN, but also dsRNA-mediated mitochondrial apoptosis.

**IMPORTANCE** Responsible for severe hemorrhagic disease in domestic pigs and wild boar, classical swine fever is recognized by the World Organisation for Animal Health (OIE) and European Union as a notifiable disease of economic importance. Persistent infection, immunotolerance, and early dissemination of the virus at local sites of infection have been linked to the antagonism of type I IFN induction by N^pro^. This protein may further contribute to these phenomena by antagonizing the induction of dsRNA-mediated apoptosis. Ultimately, apoptosis is an important innate mechanism by which cells counter viruses at local sites of infection, thus preventing wider spread and dissemination within the host, potentially also contributing to the onset of persistence. Elucidation of the mechanism by which N^pro^ antagonizes the apoptotic response will help inform the development of rationally designed live-attenuated vaccines and antivirals for control of outbreaks in typically CSFV-free countries.

## Abstract (precis)

Responsible for severe hemorrhagic disease in domestic pigs and wild boar, classical swine fever is recognized by the World Organisation for Animal Health (OIE) and European Union as a notifiable disease of economic importance. Persistent infection, immunotolerance, and early dissemination of the virus at local sites of infection have been linked to the antagonism of type I IFN induction by N^pro^.

## INTRODUCTION

Classical swine fever virus (CSFV), a pestivirus within the *Flaviviridae* family of positive-sense RNA viruses, is the causative agent of classical swine fever (CSF), a notifiable disease of domesticated pigs and wild boar. Recent and historic outbreaks have been associated with significant economic losses, and concurrently animal welfare is severely affected (1, 2). CSFV virulence and clinical outcome are multifactorial, being both age and strain dependent (2, 3). Infection of piglets less than 12 weeks of age manifests as an acute disease associated with severe leukopenia, fever, hemorrhagic disease, and a host of neurological complications (e.g., ataxia and convulsions), and death follows 1 to 3 weeks later. Disease is less acute in older pigs, often resulting in chronic infection, a phenomenon also observed prenatally in piglets infected 50 to 70 days into gestation (4). Paradoxically, chronic and prenatal infection is always lethal, while recovery from acute infection is possible (2). Together, these observations suggest a complex interplay between the virus and the host immune system.

As with interferon (IFN), apoptosis of infected cells ultimately serves as yet another mechanism by which the intracellular innate immune system is able to counter the viruses at the local site of infection and prevent their wider dissemination within the host (5). Leukopenia in CSFV-infected pigs is thought to occur as a consequence of cell death (6, 7); however, these cells rarely contain virus (8). Taking into account the high titers of virus detected in acutely infected pigs, infected cells resistant to virus-induced apoptosis likely have a role to play in determining the overall clinical outcome (9, 10).

Apoptosis is an orderly program of cell death employed by multicellular organisms to eliminate damaged, aberrant, or infected cells (11). Intracellular stimuli such as DNA damage utilize a mitochondrial pathway of cell death regulated by Bcl-2 family proteins that serves to trigger release of cytochrome *c* from the mitochondria into the cytosol (12). Subsequently, cytochrome *c* associates with Apaf-1 to form a heptametric complex called the apoptosome, enabling the cleavage of caspase-9 and the effector caspases 3 and 7 (13). Death receptor-mediated cell death is triggered in response to death factor ligands of the tumor necrosis factor (TNF) family (TNF-α, FasL, and TRAIL). Upon ligand binding, a death-inducing signaling complex (DISC) is formed, cleaving caspase-8, which can either cleave effector caspases directly or cleave Bid (tBid) to activate mitochondrial apoptosis (14). Viral antagonism of apoptosis is well documented: African swine fever virus A179L achieves this by directly binding tBid and Bax (15, 16), while vFLIP of gammaherpesviruses prevents interaction of caspase-8 with the DISC (17). Homology of viral proteins with host antiapoptotic factors is often responsible for this antagonism.

The CSFV genome encodes four structural and seven nonstructural proteins that are initially translated as a single polyprotein (18). N^pro^, a cysteine autoprotease, undergoes autocatalytic cleavage from the polyprotein (19, 20) and has been demonstrated to interact with proteins in order to modulate the intracellular innate immune response comprised primarily of type I interferon (IFN-α/β) and apoptosis. N^pro^ interacts with interferon regulatory factor 3 (IRF3), resulting in its proteasomal degradation and the elimination of host cell capacity to induce IFN in response to the pathogen-associated molecular pattern (PAMP) double-stranded RNA (dsRNA), a replication intermediate of RNA viruses (21, 22). The ability of N^pro^ to antagonize dsRNA-mediated apoptosis is, however, not well characterized, and the mechanism remains to be properly elucidated (23, 24). In addition, the pathways through which agonists such as dsRNA induce apoptosis in porcine cell lines routinely used to study CSFV require examination.

Herein, we confirm the ability of N^pro^ to inhibit dsRNA-mediated apoptosis and also show that N^pro^ is able to antagonize Sendai virus (SeV)-mediated apoptosis. Gene-edited PK-15 cell lines were used to show the dsRNA-sensing pathogen recognition receptors (PRRs) TLR3 and RIG-I specifically mediated apoptotic responses to poly(I·C) and SeV, respectively. We demonstrate that CSFV N^pro^’s interaction with porcine IRF3 is responsible not only for the antagonism of IFN induction but also the innate apoptotic response and is mediated by an inhibition of the IRF3-dependent mitochondrial translocation of Bax, a proapoptotic Bcl-2 family protein.

## RESULTS

### N^pro^ antagonizes poly(I·C)- and Sendai virus-mediated apoptosis in PK-15 cells.

**FIG 1:**
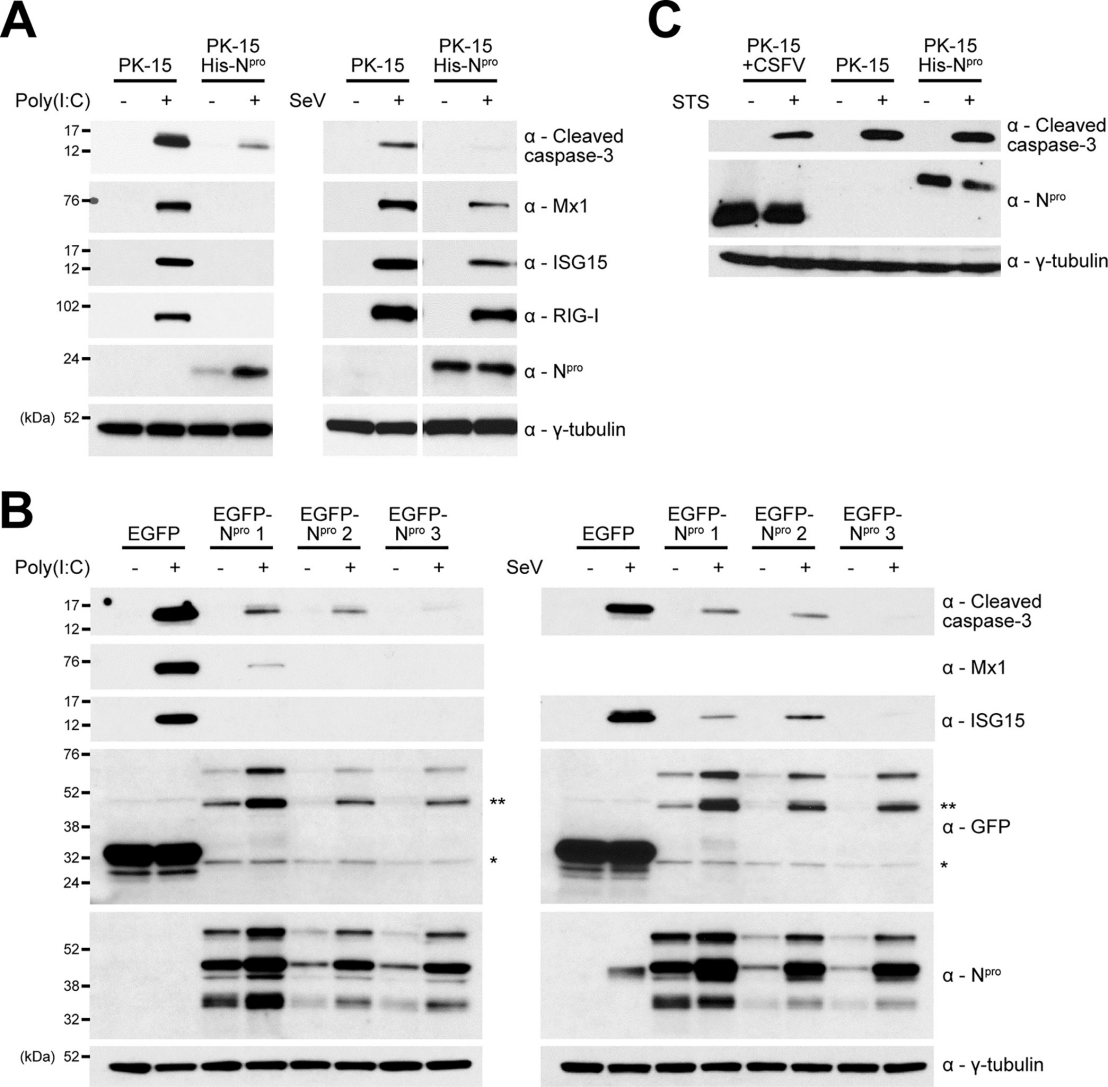
N^pro^ antagonizes poly(I·C)- and Sendai virus-mediated apoptosis in PK-15 cells. (A) Twelve-well plates were seeded with PK-15 cells or PK-15 cells stably expressing His-N^pro^ or (B) PK-15 cells stably expressing EGFP (*) or EGFP-N^pro^ (**) and treated with poly(I·C) or SeV. Eighteen hours posttreatment, whole-cell lysates were prepared and analyzed by Western blotting using polyclonal Abs recognizing ISG15, N^pro^, or GFP and MAbs recognizing cleaved caspase-3, Mx1, or RIG-I as indicated. (C) PK-15 cells, PK-15 cells stably expressing His-N^pro^, or PK-15 cells infected (MOI of 0.5) with CSFV Alfort for 24 h were treated with STS. Eight hours posttreatment, whole-cell lysates were prepared and analyzed by Western blotting using a polyclonal Ab recognizing N^pro^ and a MAb recognizing cleaved caspase-3 as indicated. (A to C) A MAb recognizing γ-tubulin was used to determine relative protein concentrations. Experiments were repeated on at least two separate occasions.

To confirm previous reports of N^pro^’s ability to antagonize dsRNA-mediated apoptosis, a porcine kidney cell line (PK-15) stably expressing His-tagged N^pro^ was treated with poly(I·C) before whole-cell lysates were examined by Western blotting for cleaved caspase-3, a terminal indicator of apoptosis. As expected, Western blot analysis showed that caspase-3 was cleaved in parental PK-15 cells treated with poly(I·C) (Fig. 1A). In contrast, the His-N^pro^ cell line exhibited a comparatively reduced level of cleaved caspase-3 following poly(C) treatment, confirming antagonism of the innate apoptotic response (Fig. 1A). As expected for cell lines that showed reduced levels of IRF3, the interferon-stimulated gene (ISG) products Mx1, ISG15, and RIG-I were not upregulated in N^pro^-expressing lines following the treatment (Fig. 1A). Poly(I·C) is thought to be an agonist of TLR3-mediated signaling when added to cell culture media. The His-N^pro^ cell line was next treated with Sendai virus (SeV; Cantell strain), a reported agonist of RIG-I-mediated signaling. Similar to that observed for poly(I·C) treatment (Fig. 1A), Western blot analysis of whole-cell lysates confirmed that SeV was able to induce the cleavage of caspase-3 in control PK-15 cells, but comparatively lower levels of caspase-3 cleavage were observed for the His-N^pro^ cell line (Fig. 1A). Subsequent analyses of ISG levels in the respective whole-cell lysates showed that SeV treatment induced the expression of Mx1, ISG15, and RIG-I in the control PK-15 cells and to a comparatively lower level in the His-N^pro^ cell line, demonstrating N^pro^’s ability to anatgonize SeV-induced ISG upregulation (Fig. 1A).

Using lentivirus, we developed PK-15 cell lines stably expressing enhanced green fluorescent protain (EGFP)-tagged N^pro^ to further validate these observations. Indeed, Western blot analyses of three individual cell lines expressing EGFP-tagged N^pro^ confirmed their ability to anatagonize poly(I·C)- and SeV-mediated cleaved caspase-3 production and ISG upregulation in comparison to a control EGFP-expressing cell line (Fig. 1B).

As we have adopted cleaved caspase-3 as our primary readout for apoptosis, N^pro^ and CSFV were assessed for their capacity to antagonize apoptosis mediated by staurosporine (STS), an agonist that triggers caspase-3 cleavage through pathways independent of those typically associated with dsRNA signaling (25, 26). In agreement with past observations (24), when CSFV-infected PK-15 cells or a PK-15 cell line stably expressing His-N^pro^ were treated with STS, levels of cleaved caspase-3 were comparable to that observed in uninfected control cells (Fig. 1C).

### Type I IFN amplifies poly(I·C)-mediated apoptosis in PK-15 and SK6 cells but is not essential.

**FIG 2:**
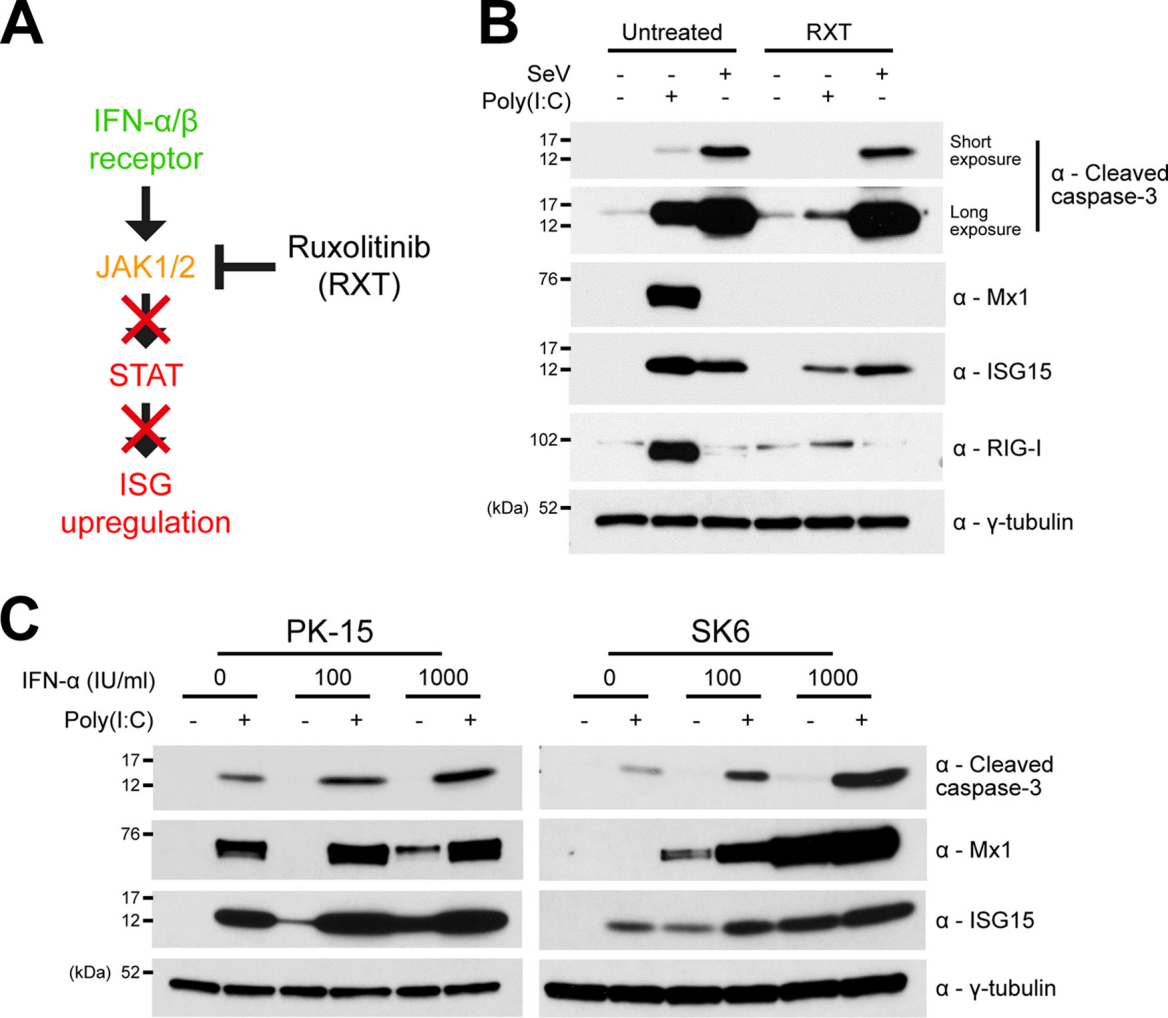
Type I IFN amplifies poly(I·C)-mediated apoptosis but is not essential for its induction. (A) Schematic representation of RXT inhibition of JAK/STAT-mediated IFN response. (B) Twelve-well plates were seeded with PK-15 cells and treated with poly(I·C) or SeV in the presence or absence of JAK-STAT inhibitor RXT. Eighteen hours posttreatment, whole-cell lysates were prepared and analyzed by Western blotting using a polyclonal Ab recognizing ISG15 or MAbs recognizing cleaved caspase-3, Mx1, RIG-I, or GFP as indicated. A MAb recognizing γ-tubulin was used to determine relative protein concentrations. (C) Twelve-well plates were seeded with PK-15 and SK6 cells and treated with increasing concentrations of porcine IFN-α in the presence or absence of poly(I·C). Eighteen hours posttreatment, whole-cell lysates were prepared and analyzed by Western blotting as in panel A. Experiments were repeated on at least two separate occasions.

Since we as well as others have observed N^pro^’s clear antagonism of poly(I·C)-mediated IFN-induction and ISG upregulation (21–23, 27), we wanted to establish whether IFN had any role in the induction of apoptosis in response to each agonist—poly(I·C) and SeV. To do this, wild-type (WT) PK-15 cells were treated with either poly(I·C) or SeV in the presence of the JAK-STAT inhibitor ruxolitinib (RXT [Fig. 2A]), and whole-cell lysates were then analyzed by Western blotting for cleaved caspase-3. Interestingly, a large reduction in cleaved caspase-3 was observed in comparison to cells treated with poly(I·C) in the absence of RXT (Fig. 2B). However, the levels of cleaved caspase-3 in cells treated with SeV were unaffected by the presence of RXT. To confim RXT treatment had efficiently blocked IFN signaling following poly(I·C) treatment, lysates were then analyzed for expression of the ISG proteins Mx1, ISG15, and RIG-I: as expected, the upregulation of Mx1 and RIG-I was inhibited in the presence of RXT, while ISG15 upregulation was only partially antagonized since IRF3 can bind directly to its promoter (28–30).

In order to further elucidate the impact that IFN has on poly(I·C)-mediated apoptosis, PK-15 and SK6 cells were treated with poly(I·C) in the presence of increasing quantities of porcine IFN-α (0, 100, and 1,000 IU/ml). For both the treated PK-15 and SK6 cells, subsequent Western blot analyses revealed a positive correlation between the quantity of IFN-α used and the observed level of cleaved caspase-3 (Fig. 2C). The increase in cleaved caspase-3 was most noticable with SK6 cells, a cell line known to be incapable of producing endogenous type I IFN (27). Importantly, IFN-α treatment alone was incapable of triggering levels of caspase-3 cleavage comparable to that observed in cells treated with poly(I·C) alone.

### Poly(I·C)- and SeV-mediated apoptosis is dependent on TLR3 and RIG-I signaling pathways converging on IRF3 in PK-15 cells.

**FIG 3:**
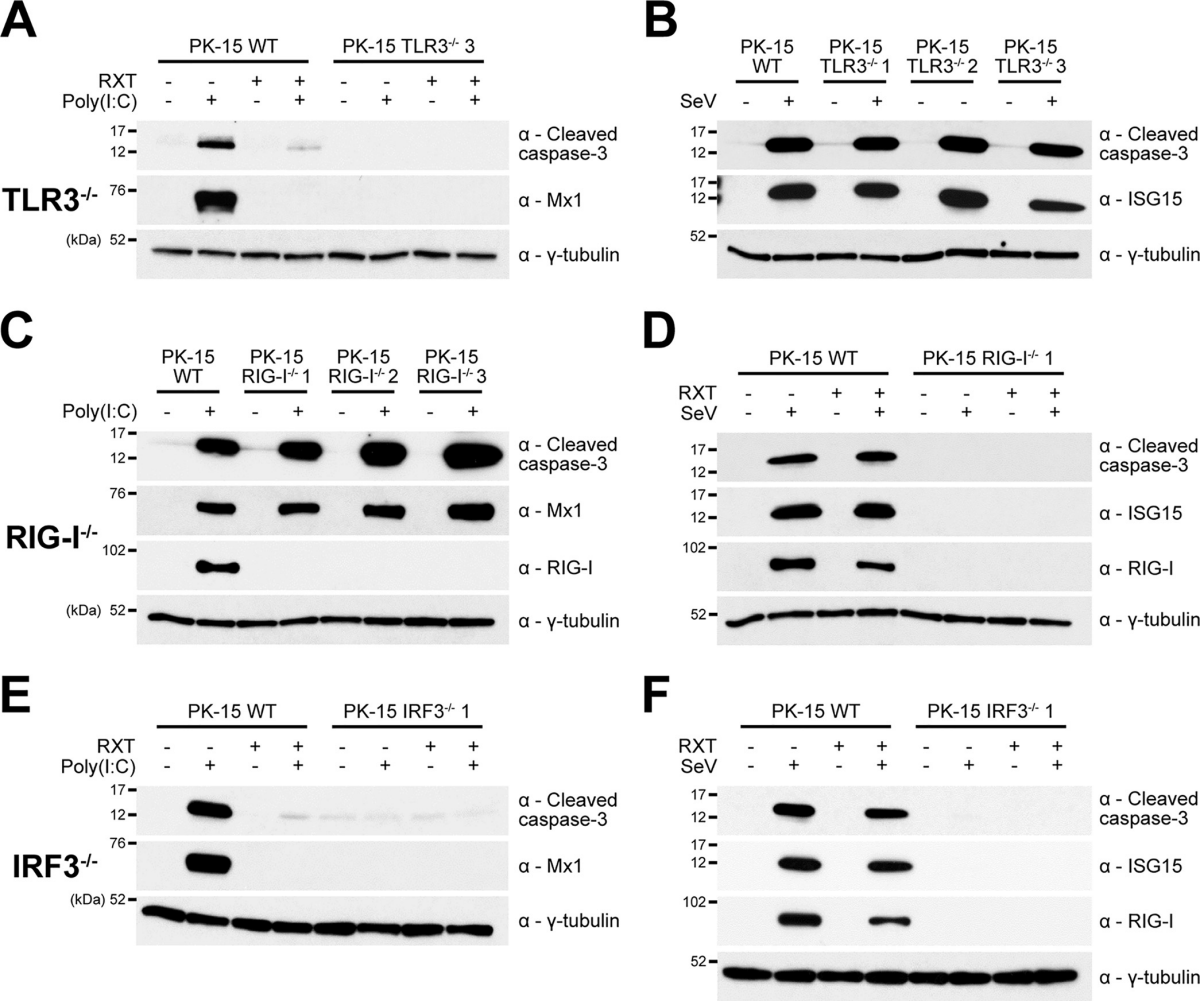
Poly(I·C) and SeV induce apoptosis through TLR3/IRF3 and RIG-I/IRF3 signaling pathways, respectively. (A to F) Twelve-well plates were seeded with WT PK-15 and knockout PK-15 cell lines (TLR3^−/−^, RIG-I^−/−^, and IRF3^−/−^) and treated with poly(I·C) (A, C, and E) or SeV (B, D, and F) in the presence or absence of RXT. Eighteen hours posttreatment, whole-cell lysates were harvested and analyzed by Western blotting using a polyclonal Ab recognizing ISG15 and MAbs recognizing cleaved caspase-3, Mx1, or RIG-I, as indicated. A MAb recognizing γ-tubulin was used to determine relative protein concentrations. Experiments were repeated on at least two separate occasions.

In order to identify the innate cell signaling pathways through which poly(I·C) and SeV induce apoptosis in PK-15 cells and to help elucidate the mechanism that N^pro^ uses to achieve the observed antagonism of apoptosis, PK-15 cell lines were developed that had been gene edited to knock out the expression of TLR3 (TLR3^−/−^) and RIG-I (RIG-I^−/−^). For each targeted gene, individual cell lines were generated and validated by PCR, sequencing, and when a suitable antibody (Ab) was available, by Western blotting. Each cell line was screened by Western blotting for responsiveness to poly(I·C) and SeV in the presence or absence of RXT. Cleaved caspase-3 was undetectable in TLR3^−/−^ cells following poly(I·C) treatment, whereas the cleavage of caspase-3 induced by SeV infection was unaffected by the loss of TLR3 (Fig. 3A and B). In contrast, RIG-I^−/−^ cell lines displayed the opposite phenotype, namely, loss of cleavage of capsase-3 in response to SeV but normal cleavage in response to poly(I·C) (Fig. 3C and D). These results confirmed that poly(I·C) and SeV trigger apoptosis in PK-15 cells specifically via TLR-3 and RIG-I, respectively.

Since TLR3 and RIG-I signaling pathways are classically known to converge on IRF3 to activate the IFN-β promoter, we next investigated if IRF3 is also required for the induction of apoptosis. To facilitate this, PK-15 cell lines gene edited to knock out IRF3 (IRF3^−/−^) were prepared and validated (B. Jackson, S. Hardy, E. Reid, B. Charleston, S. Goodbourn, and J. Seago, submitted for publication). Interestingly, no cleaved caspase-3 was observed when IRF3^−/−^ PK-15 cells were treated with either poly(I·C) or SeV (Fig. 3E and F). In each case, absence of caspase-3 cleavage was associated with an absence of ISG upregulation, highlighting that the pathways responsible for IFN induction are also responsible for induction of the innate apoptotic response to these antagonists. The presence of RXT had no observable effect on caspase-3 cleavage in the knockout PK-15 cell lines.

### Bax directly mediates apoptosis in a manner that depends upon transcription-independent functions of IRF3.

**FIG 4:**
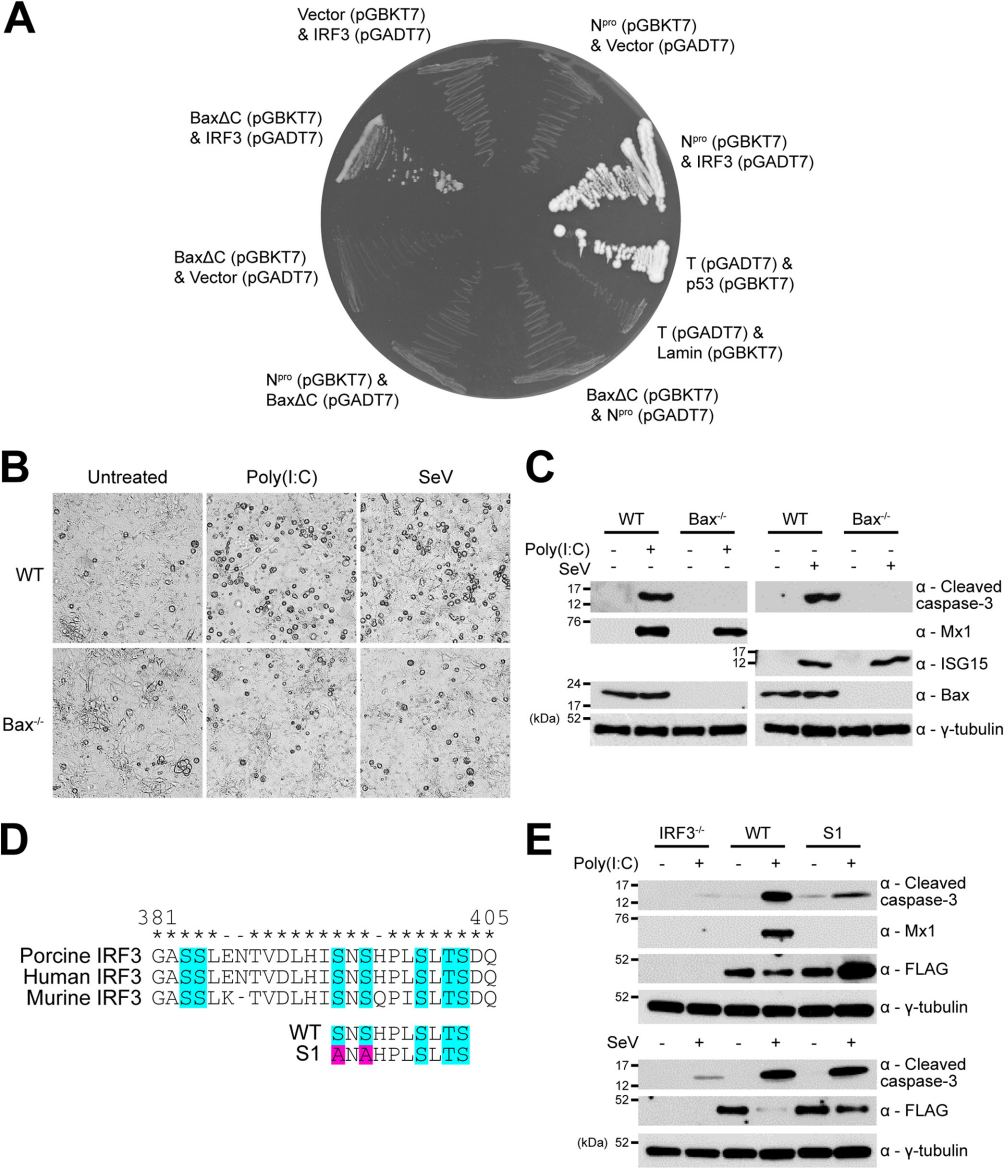
Bax directly mediates apoptosis in a manner that relies upon transcription-independent functions of IRF3. (A) Yeast strains cotransformed with plasmids expressing the indicated proteins fused to either the GAL4 DNA-binding domain (in pGBKT7) or activation domain (in pGADT7) were cultured on dropout media to identify interactions. Cotransfection of plasmids encoding N^pro^, BaxΔC, or IRF3 with the reciprocal plasmid vector (pGBKT7 or pGADT7) served as negative interaction controls. Cotransfection of the large T antigen (T) and p53 or T and lamin served as positive and negative system controls. (B and C) Twelve-well plates were seeded with WT and Bax^−/−^ PK-15 cell lines and treated with poly(I·C) or SeV. Eighteen hours posttreatment, whole-cell lysates were (B) imaged and then (C) harvested and analyzed by Western blotting using polyclonal Abs recognizing ISG15 or Bax and MAbs recognizingcleaved caspase-3 or Mx1. (D) Alignments of porcine, human, and murine IRF3 protein sequences implicated in transcriptional activity (turquoise) were performed in MEGA7. Mutations (pink) were designed in porcine IRF3 (poIRF3) to generate N-terminal FLAG-tagged WT and S1 mutant (S394A S396A) poIRF3 fusion proteins. Conserved (*) and nonconserved (−) residues are indicated. (E) Pools of IRF3^−/−^ PK-15 cells expressing WT or S1 mutant FLAG-tagged IRF3 were prepared and treated as previously detailed (B and C). Western blot analysis was performed using a polyclonal Ab recognizing the FLAG epitope (DYKDDDDK) and MAbs recognizing cleaved caspase-3 or Mx1. (C and E) A MAb recognizing γ-tubulin was used to determine relative protein concentrations. Experiments were repeated on at least two separate occasions.

Having shown that IRF3 is required for the induction of TLR3- and RIG-I-mediated apoptotic responses, it was important to determine the mechanism of IRF3 function. IRF3 has been reported to play a role in a transcription-independent pathway of apoptosis that relies upon an interaction with the proapoptotic protein Bax and its subsequent translocation to the mitochondrial membrane in murine and human cells (31–33). To establish whether porcine IRF3 interacts with porcine Bax, the yeast two-hybrid (Y-2-H) system was employed. In agreement with previous reports (34–36), we found full-length Bax expression toxic in yeast, but a truncated mutant lacking the C-terminus transmembrane domain (BaxΔC) exhibited less toxicity (37–39) and was used to successfully confirm the interaction (Fig. 4A). Further Y-2-H analyses confirmed N^pro’^s ability to interact with porcine IRF-3, but no direct interaction between N^pro^ and BaxΔC was observed (Fig. 4A). To validate the physiological significance of this interaction, additional PK-15 cell lines were developed that had been gene edited to knock out the expression of Bax (Bax^−/−^); successful knockout was confirmed by Western blotting. Subsequently, Bax^−/−^ cells were screened by live-cell bright-field microscopy (Fig. 4B) and Western blotting (Fig. 4C) for their responsiveness to poly(I·C) and SeV, identified as specific ligands for TLR3 and RIG-I respectively, in PK-15 cells (Fig. 3). Unedited PK-15 cells displayed significant rounding and detachment following both poly(I·C) and SeV treatments, indicative of apoptosis. In contrast, Bax^−/−^ PK-15 cells remained largely unchanged following each treatment (Fig. 4B). Cleaved caspase-3 was undetectable in Bax^−/−^ cells following each treatment, while Mx1 and ISG15 were detected at comparable levels in both unedited and Bax^−/−^ PK-15 cells (Fig. 4C). These results confirmed that IRF3-mediated apoptosis is Bax dependent.

IRF3 is best known for its function as a transcription factor, mediating the upregulation of not only type I IFN but also a small subset of “IFN-independent” ISGs (28–30). In order to determine whether apoptosis requires IRF3 transcriptional activity, IRF3^−/−^ PK-15 cells stably expressing a FLAG-tagged transcriptionally inactive IRF3 mutant (S394A S396A) termed “S1” (31, 40) were generated using lentivirus (Fig. 4D). These serine residues are potentially required for the interaction between IRF3 and CREB-binding protein (CBP), a prerequisite for the binding of IRF3 to gene promoters (41–43) and are also highly conserved across multiple species (Fig. 4D). In mice, the S1 mutations eliminate the ability of IRF3 to stimulate transcription while preserving its proapoptotic functions (31). As a control, an IRF3^−/−^ PK-15 cell line stably expressing FLAG-tagged WT IRF3 was also generated. Both cell lines were then subjected to poly(I·C) treatment, and whole-cell lysates were examined by Western blotting for the presence of Mx1 as an indicator of IRF3 transcriptional activity, as well as cleaved caspase-3 to determine the induction of apoptosis. Mx1 was undetectable in S1 samples following poly(I·C) treatment, confirming the loss of transcriptional activity and the ability to induce type I IFN. In contrast, Mx1 was observed in the corresponding WT samples. However, cleaved caspase-3 was observed in both the WT and, to a lesser extent, the S1 IRF3 samples (Fig. 4E), confirming that the transcriptionally inactive S1 mutant could still mediate apoptosis. Similar experiments using SeV treatment led to comparable levels of caspase-3 cleavage (Fig. 4E) in WT and S1 IRF3-expressing IRF3^−/−^ PK-15 cell lines. Together, these results confirmed that IRF3 mediates a Bax-dependent pathway of apoptosis, even when devoid of its ability to act as a transcription factor.

### N^pro^ blocks poly(I·C)- and Sendai virus-mediated mitochondrial localization of Bax.

In the present work, IRF3 has been shown to coordinate a TLR3- and RIG-I-mediated Bax-dependent pathway of apoptosis independent of its activity as a transcription factor, supporting previous observations made by Chattopadhyay et al. (31–33). However, the exact nature of porcine Bax’s role in this process remains to be determined. CSFV N^pro^ has previously been shown to antagonize poly(I·C)-mediated mitochondrial release of cytochrome *c* and caspase-9 cleavage (24). Furthermore, N^pro^’s ability to target IRF3 for ubiquitin-dependent proteasomal degradation has been well documented (21). We therefore decided to investigate if Bax can localize to the mitochondrial membrane following the induction of apoptosis in the presence of N^pro^ and in the absence of IRF3.

**FIG 5:**
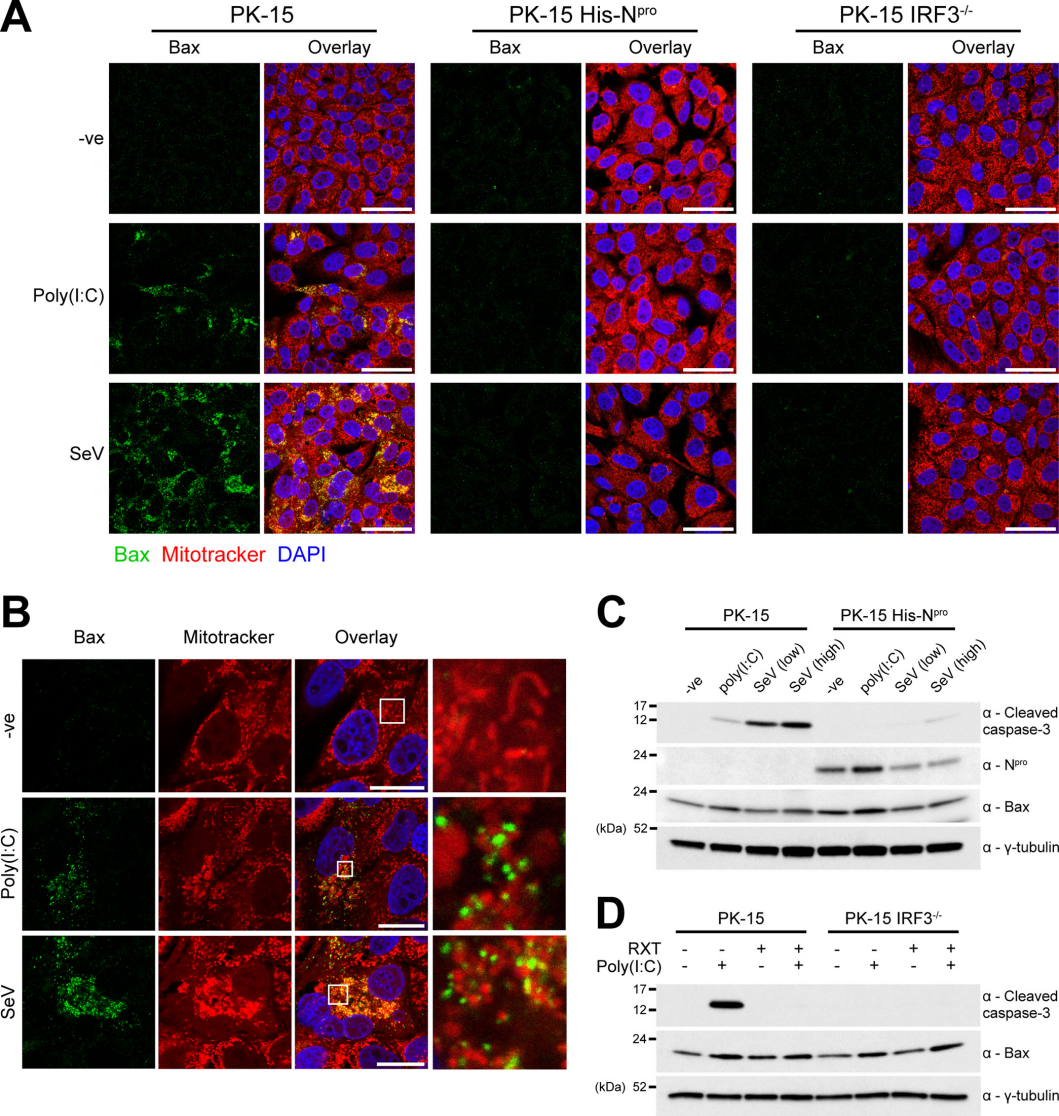
N^pro^ blocks poly(I·C)- and Sendai virus-mediated mitochondrial localization of proapoptotic Bax. (A) WT, IRF3^−/−^, and His-N^pro^-expressing PK-15 cells were treated with poly(I·C) or SeV in the presence of 100 µM caspase inhibitor Z-VAD(OMe)-FMK (Bachem). Eighteen hours posttreatment, cells were treated with MitoTracker and analyzed by immunofluorescence using a polyclonal Ab recognizing Bax. Nuclei are stained blue with DAPI. Scale bars represent 45 μm. (B) Immunofluorescence images of single cells were collected from the experiment detailed in panel A. Scale bars represent 20 μm. (C and D) Whole-cell lysates prepared from replicate samples of panel A were analyzed by Western blotting using polyclonal Abs recognizing Bax or N^pro^ and a MAb recognizing cleaved caspase-3. A MAb recognizing γ-tubulin was used to determine relative protein concentrations. Experiments were repeated on at least two separate occasions.

Experiments using PK-15 cells and immunofluorescence confocal microscopy were performed to confirm the localization of endogenous Bax to the mitochondria following exposure to the agonists poly(I·C) and SeV. In both poly(I·C)- and SeV-treated PK-15 cells, Bax localization was undetectable prior to treatment, in agreement with published literature (31–33). However, following treatment with either poly(I·C) or SeV, Bax was detectable, appearing as distinct, condensed puncta that colocalized with the mitochondrial membrane, but did not appear to have been internalized (Fig. 5A and B). In PK-15 cells that had been treated with either agonist, the mitochondria exhibited a condensed morphology characteristic of apoptosis. Bax localization was also investigated in PK-15 cell lines stably expressing N^pro^ and in the PK-15 IRF3^−/−^ cell lines. While a large proportion of WT PK-15 cells displayed mitochondrial localization of Bax, there was a complete absence in both the N^pro^ and IRF3^−/−^ PK-15 cell lines tested (Fig. 5A). These experiments were performed in the presence of Z-VAD(OMe)-FMK (Bachem), an inhibitor of the effector caspases, in order to maximize the number of cells for visualization by immunofluorescence following treatment with each apoptosis agonist. Collectively, these observations highlight the role of Bax in IRF3-medated apoptosis and confirm its antagonism by N^pro^.

Having shown that the presence of N^pro^ inhibited the mitochondrial localization of Bax, experiments were conducted to determine whether N^pro^ can also modulate Bax expression in a similar manner to that observed for IRF3. Western blot analysis of whole-cell lysates prepared from untreated WT and N^pro^-expressing PK-15 cells indicated that Bax is not targeted by N^pro^ for degradation (Fig. 5C). Interestingly, poly(I·C) treatment led to an increase in the level of N^pro^ protein compared to that in untreated cells, raising a possibility of stabilization in the presence of a target protein.

### Antagonism of SeV-mediated apoptosis in CSFV-infected cells is dependent on the expression of N^pro^.

**FIG 6:**
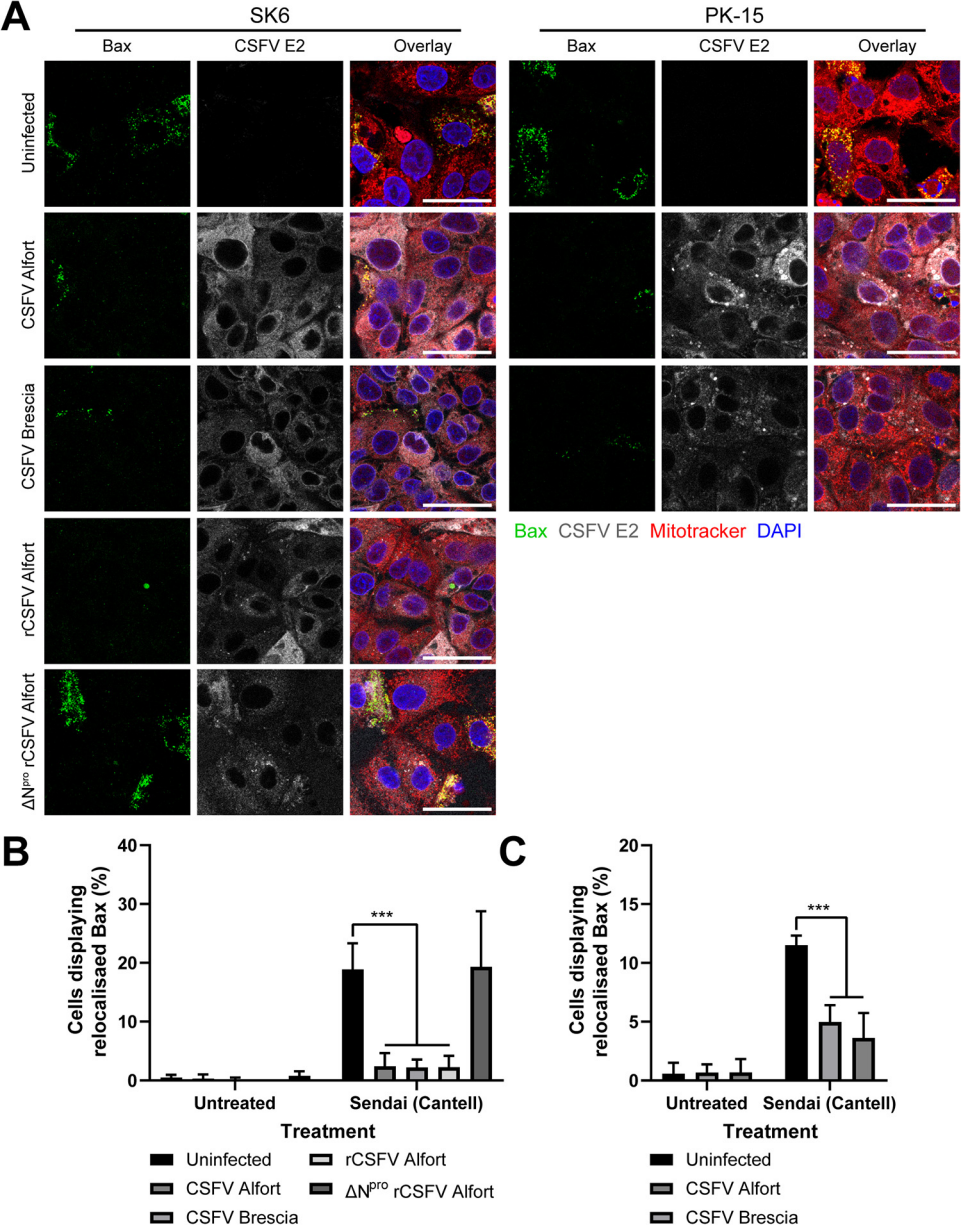
Antagonism of SeV-mediated apoptosis in CSFV-infected cells is dependent on the expression of N^pro^. (A) WT PK-15 and SK6 cells were infected (MOI of 0.2) with CSFV Alfort, CSFV Brescia, rCSFV Alfort, or ΔN^pro^ rCSFV Alfort as indicated for 72 h and then treated with SeV. Eighteen hours posttreatment, cells were treated with MitoTracker and analyzed by immunofluorescence staining using a polyclonal Ab recognizing Bax and a MAb recognizing CSFV E2. Nuclei are stained blue with DAPI. (B and C) The percentage of cells displaying Bax localization to the mitochondria following each treatment was then quantified. ***, *P* < 0.001. Experiments were repeated on at least two separate occasions.

In order to investigate whether CSFV infection has the same antagonistic effect on apoptosis as stably expressed N^pro^, SK6 cells were infected (multiplicity of infection [MOI] of 0.2) with either CSFV Alfort, CSFV Brescia, a recombinant CSFV (rCSFV) Alfort strain, or an rCSFV Alfort strain with N^pro^ deleted (ΔN^pro^); infections were allowed to continue until most cells had been infected (as determined by CSFV E2 expression). Infected cells were then treated with SeV prior to analysis by immunofluorescence microscopy (Fig. 6A). Due to the inability of ΔN^pro^ rCSFV Alfort to efficiently replicate in PK-15 cells (27), SK6 cells were used instead as they lack the capacity to produce type I IFN and are sensitive to dsRNA-mediated apoptosis (23, 27). The SK6 cells that were infected with either CSFV Alfort, CSFV Brescia, or rCSFV Alfort prior to SeV treatment displayed significantly reduced Bax localization to the mitochondria (*P* < 0.001); however, those infected with ΔN^pro^ rCSFV Alfort displayed levels of localization comparable to that of uninfected control cells (Fig. 6A and B). CSFV was not assessed for its capacity to antagonize poly(I·C)-mediated apoptosis as the ΔN^pro^ virus still encodes the soluble and secretable endoribonuclease E^rns^, which acts as a scavenger receptor for dsRNA (44, 45); a double mutant ΔN^pro^ ΔE^rns^ virus was unavailable.

We subsequently infected (MOI of 0.2) PK-15 cells with either CSFV Alfort or CSFV Brescia in order to validate the capacity of CSFV infection to antagonize induction of apoptosis in a more relevant, IFN-competent cell line. As with the SK6 infections, PK-15 cells infected with either CSFV Alfort or CSFV Brescia displayed reduced Bax localization to the mitochondria (*P* < 0.001) (Fig. 6A and C). In summary, these results confirm that CSFV is indeed capable of antagonizing SeV-mediated mitochondrial Bax localization in multiple porcine kidney endothelial cell lines, suggesting a clear capacity to antagonize induction of apoptosis dependent on the expression of N^pro^.

## DISCUSSION

**FIG 7:**
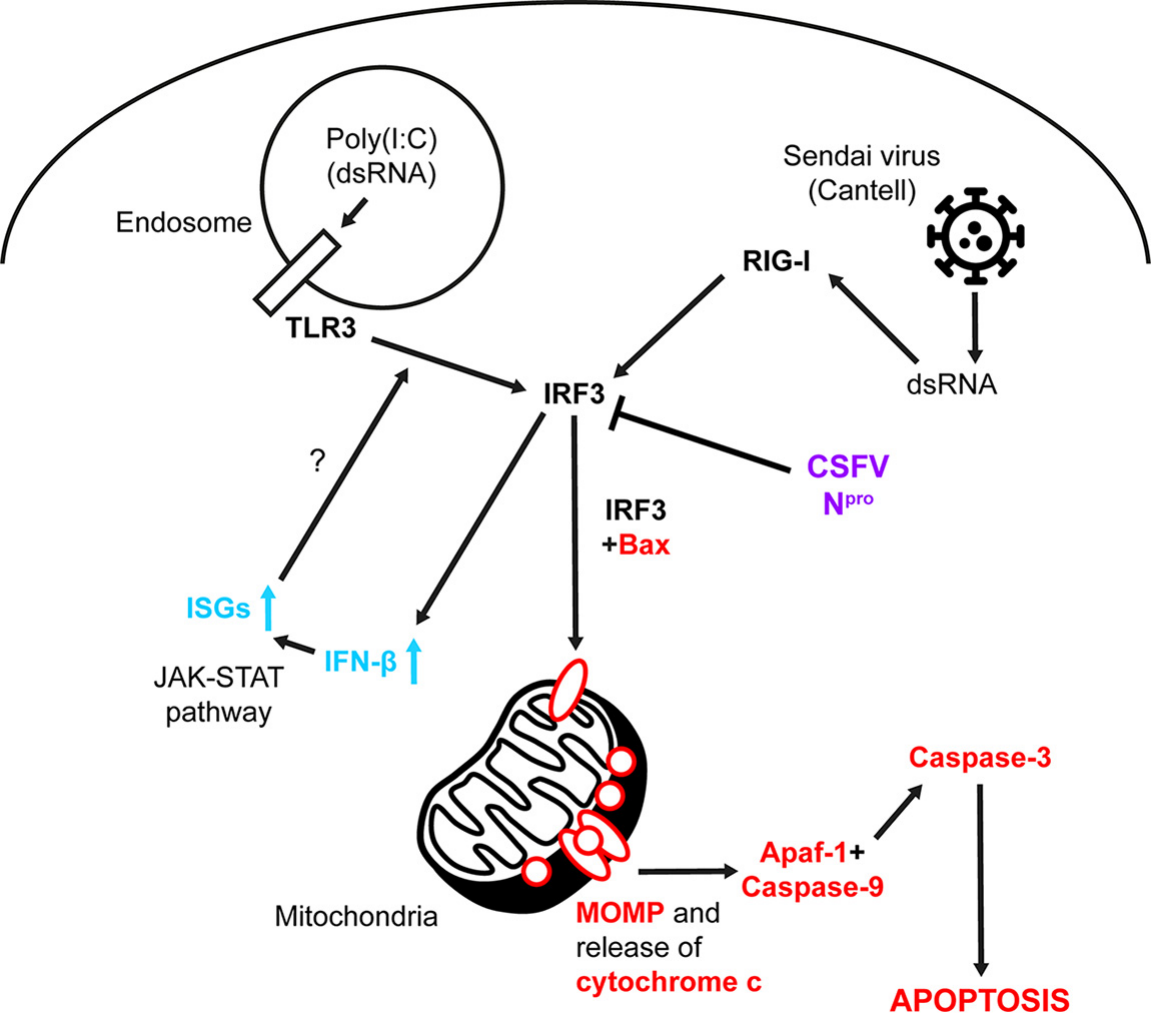
Model of TLR3- and RIG-I-mediated apoptosis and its antagonism by CSFV N^pro^. Upon stimulation with poly(I·C) and SeV, TLR3 and RIG-I initiate apoptosis in an IRF3-dependent manner, independent of its functions as a transcription factor and characterized by mitochondrial relocalization of Bax and activation of caspase-3. IRF3 also triggers induction of IFN-β and IFN-dependent and -independent upregulation of ISGs which might amplify the TLR3/IRF3 signaling axis. CSFV N^pro^ (purple), apoptotic signaling (red), IFN signaling (blue), and uncertain or inferred (?) pathways are indicated.

Generated as replication intermediates of the RNA virus genome, dsRNA triggers the induction of innate responses such as type I IFN and apoptosis. The apoptosis triggered by dsRNA as a consequence of infection is thought to have a protective role, serving to limit further virus dissemination within the host (5). When expressed stably and during infection, CSFV N^pro^ has been shown to antagonize both of these responses (21–24, 27). While N^pro^’s putative interaction with IRF3 has been identified as the source of IFN antagonism, the mechanism by which N^pro^ antagonizes the induction of dsRNA-meditated apoptosis has yet to be identified. Using a combination of CRISPR-Cas9 gene-editing technology and confocal microscopy, we report that in porcine kidney endothelial cells, IRF3 coordinates the induction of RIG-I- and TLR3-mediated apoptosis in an IRF3-dependent IFN-independent manner, culminating in the localization of proapoptotic Bax to the mitochondrial membrane and induction of caspase-3 cleavage, a key hallmark of apoptosis (Fig. 7).

Initially we identified the pathways that N^pro^ is able to antagonize and thereafter identified the PRRs through which agonists were sensed in order to elucidate the mechanism of apoptosis inhibition used by N^pro^. In addition to antagonizing apoptosis mediated by poly(I·C), a reported TLR3 agonist and dsRNA homolog, we report that N^pro^ expressed stably in cell culture and during infection can also antagonize SeV-mediated apoptosis. This finding was interesting since SeV copy-back defective interfering (cbDI) RNA is widely reported to be an agonist of the RIG-I signaling pathway (46–48) and showed that N^pro^ is capable of targeting proapoptotic signaling triggered by multiple pathways. CRISPR-Cas9 knockouts of both TLR3 and RIG-I subsequently confirmed them to be essential in PK-15 cells for the induction of caspase-3 cleavage in response to poly(I·C) and SeV, respectively, and found IRF3 to be indispensable in each case.

Since N^pro^’s putative interaction with IRF3 and its consequent antagonism of type I IFN induction are well published, we intended to determine whether IFN has any role in the caspase-3 cleavage observed in response to the TLR3 and RIG-I agonists poly(I·C) and SeV. Pharmacological inhibition of the JAK-STAT pathway using RXT and subsequent treatment of cells with porcine IFN-α revealed the apoptosis mediated by poly(I·C), but not SeV, to be amplified, while IFN-α alone appeared to cause no detectable caspase-3 cleavage. The type I IFN response is required for the upregulation of a diverse range of ISGs, a number of which are proapoptotic. In light of this, it is possible that components of the TLR3 signaling pathway might be upregulated by type I IFN, thus explaining the observed amplification of caspase-3 cleavage. Shaw et al. reported upregulation of TLR3, caspase-8, Noxa, and TRAIL expression in *ex vivo* porcine skin fibroblast cultures following IFN treatment (49), while Renson et al. found elements of the Fas and TRAIL signaling pathways to be upregulated in uninfected bystander peripheral blood mononuclear cells (PBMCs) during *in vivo* infection with a related pestivirus, bovine viral diarrhea virus (BVDV) (50). Direct amplification of poly(I·C)-mediated apoptosis by IFN-α has also been described (51); however, the capacity of each to modulate the innate apoptotic response likely varies, depending on tissue and cell type. SeV encodes C-protein, an antagonist of STAT1 phosphorylation, which likely explains the apparent absence of ISG upregulation or subsequent effect from the RXT treatment (52).

The apparent importance of IRF3 in coordinating the induction of proapoptotic TLR3- and RIG-I-mediated responses proved insightful, since IRF3 has previously been implicated in the induction of a dsRNA-mediated IRF3/Bax-dependent pathway of apoptosis termed RIPA (RLR-induced IRF3-mediated pathway of apoptosis) that also culminates in cleavage of caspase-3 (31–33). Through a putative BH3-like domain, IRF3 has been shown to mediate its proapoptotic activity through a direct interaction with Bax, facilitating its localization to the mitochondrial membrane (32). In this study, we have confirmed the interaction of porcine IRF3 and Bax using the Y-2-H system, corroborating past observations (32). We have also shown that the aforementioned IRF3/Bax-dependent pathway of apoptosis is active in porcine kidney endothelial cells and shown that IRF3-mediated apoptosis is dependent on the presence of Bax and does not require IRF3’s activity as a transcription factor. Importantly, apoptosis was actively antagonized by both stable expression of N^pro^ and infection with CSFV, as seen by the absence of, or significant reduction in, mitochondrial Bax localization and associated cleavage of caspase-3. Bax staining in apoptotic cells appeared as distinct puncta associated with the mitochondria, likely corresponding to the formation of homodimeric pores in the mitochondrial outer membrane (MOM) (53–57), which have been reported to facilitate release of cytochrome *c* from the intermembrane space (IMS) (58, 59). This is in agreement with a past study which found CSFV to antagonize cytochrome *c* release and caspase-9 cleavage (24). Importantly, this localization occurred in a manner independent of Bax expression levels, lending further credence to the idea that the observed phenotype is due to IFN-independent IRF3/Bax activity.

Jefferson et al. observed that transfected CSFV N^pro^ and the related pestivirus BVDV antagonized sodium arsenate-mediated mitochondrial Bax localization (60). However, this agonist is thought to trigger apoptosis by upregulating Bax expression in a c-Jun N-terminal kinase (JNK)-dependent manner (61). Our study explored this pathway in the context of stably expressed N^pro^ and CSFV infection, utilizing authentic agonists of dsRNA signaling pathways. We have demonstrated both poly(I·C) and SeV to be relevant and authentic agonists of TLR3 and RIG-I signaling pathways that converge on IRF3 in their induction of apoptosis. The significance of TLR3- and RIG-I-mediated responses during CSFV infection was highlighted by Hüsser et al. using short hairpin RNA (shRNA) knockdown to target each (62). No observable differences in ΔN^pro^ rCSFV growth were observed in a representative Bax^−/−^ cell line in comparison to wild-type cells (data not shown). We suspect any differences were masked by the transcriptional activity of IRF3 and subsequent ISG expression.

Taken together, these results suggest that N^pro^’s interaction with IRF3 is responsible not only for antagonizing the induction of type I IFN but also the induction of IFN-independent IRF3/Bax-mediated caspase-3 cleavage and apoptosis. Ultimately, N^pro^’s antagonism of TLR3-, RIG-I-, and IRF3-mediated apoptotic responses serves as another mechanism of CSFV immune evasion, likely contributing to the establishment of infection and host persistence.

## MATERIALS AND METHODS

### Cell culture and viruses.

All cells were maintained at 37°C in 5 % CO_2_. The PK-15, SK6, and HEK-293T cell lines (obtained in-house) were maintained in Dulbecco's modified Eagle’s medium (DMEM; Thermo Fisher), 5% adult bovine serum (ABS; Selborne) demonstrated to be BVDV free and anti-BVDV antibody free, GlutaMAX (Thermo Fisher) and penicillin-streptomycin (50 IU/ml penicillin, 50 μg/ml streptomycin; Thermo Fisher). CSFV strains Alfort 187 and Brescia were kindly provided by the EU reference laboratory (Hannover, Germany). The parental infectious clone EP 98/2 derived from CSFV-strain Alfort Tübingen and the corresponding infectious clone with N^pro^ deleted, EP 96/2, were a kind gift from Gregor Meyers (FLI; Tübingen, Germany). Virus was grown in SK6 cells and isolated from washed cell pellets by freeze-thaw lysis. Immunostaining with anti-CSFV E2 antibody WH303 (APHA) (63) was used to determine the titer of viruses by 50% tissue culture infective dose (TCID_50_) in SK6 cells and used in experiments at an MOI of 0.2. Where indicated, cells were treated with the inhibitor of JAK1/2 phosphorylation ruxolitinib (RXT; Selleckchem), recombinant porcine IFN-α (R&D Systems), staurosporine (STS; Sigma), Sendai virus (SeV) Cantell strain (Charles River), and poly(I·C) (Sigma). Cells were treated with 0.5 μM RXT, 2.5 μM STS, 100 µg/ml poly(I·C), and 200 hemagglutinating units (HAU)/ml SeV, except where stated otherwise.

### Generation of cell lines stably expressing recombinant proteins using lentivirus.

CSFV Alfort 187 cDNA was cloned into the 3rd-generation lentiviral vector pLJM1-EGFP, a gift from David Sabatini (Addgene plasmid 19319) (64), to generate pLJM1-EGFP-N^pro^. WT and S1 mutant (S396A S398A) porcine IRF3 cDNAs bearing an N-terminal FLAG tag were cloned into a modified pLJM1 vector devoid of EGFP to generate pLJM1-FLAG-IRF3 and pLJM1-FLAG-IRF3-S1. Packaging plasmids (pLP1, pLP2, and pLP/VSV-G) were cotransfected into HEK-293T cells with a single pLJM1 vector to generate lentiviruses encoding EGFP, EGFP-N^pro^, WT IRF3, and S1 IRF3, respectively. Lentiviruses were added to a low-passage-number PK-15 cell culture in the presence of 2 µg/ml Polybrene (Sigma) and centrifuged at 1,000 relative centrifugal force (rcf) for 30 min. Seventy-two hours postinfection, cells were treated for a further 72 h with 3 µg/ml puromycin (Thermo Fisher) to select for transduced cells. Forty-eight hours after removal of selection, colonies of surviving cells were picked and isolated for screening and validation.

### Generation of CRISPR-Cas9 knockout cell lines.

Single guide RNAs (sgRNAs) were designed using the E-CRISP tool (http://www.e-crisp.org/E-CRISP/designcrispr.html; German Cancer Research Center) and cloned into pSpCas9n(BB)-2A-GFP (px461) and pSpCas9n(BB)-2A-Puro (px462) V2.0 plasmids encoding the D10A nickase mutant of Streptococcus pyogenes Cas9 (Cas9n) bearing puromycin and GFP selection markers, respectively (65). The sequences of the sgRNAs are shown in Table 1. These plasmids were a gift from Feng Zhang (Addgene plasmids 48140 and 62987) (65). CaCl_2_-competent JM109 Escherichia coli cells were transformed with each plasmid, which was then extracted and purified using a QIAprep Spin Miniprep kit (Qiagen). Low-passage-number PK-15 cells were cotransfected with each plasmid for 48 h, and 3-µg/ml puromycin selection (Thermo Fisher) was applied for a further 72 h. Forty-eight hours after removal of selection, colonies of surviving cells were picked and isolated for screening and validation.

**TABLE 1:** Cas9 target sequences within the coding sequence of each gene and their respective offsets

Knockout target	Sequence		Offset
	sgRNA-1 (px461)	sgRNA-2 (px462)	
IRF3	GCCGCAAGCCGTGCTTCCAA	GGAGGACTTCGGCATCTTCC	+13
TLR3	CTCCATCCAAGGTAGTAAGT	ATTTAACACCATCTCAAAGC	+1
RIG-I	GATGATGGAGATAGAGAGTC	GATGCACTTAAATCTGTCAG	+11
Bax	TTCTTGGTAGATGCATCCTG	AGCGAGTGTCTCAAGCGCAT	+4

### Western blot analysis.

Proteins were separated by SDS-PAGE (4 to 20% polyacrylamide; Thermo Fisher) and transferred to Amersham Protran 0.45-µm-pore nitrocellulose membranes. Membranes were blocked with 5 % (wt/vol) dried skimmed milk in phosphate-buffered saline (PBS) containing 0.5 % Tween 20. Anti-CSFV N^pro^ rabbit serum (DS14) was generated in-house by inoculating rabbits with the peptide KTNKQKPMGVEEPVYDATGKPLFGDPS, corresponding to N-terminal residues 11 to 37 (66). Primary monoclonal antibodies (MAbs) recognizing γ-tubulin (T6557; Sigma), Mx1 (Ab79609; Abcam), RIG-I (sc-376845; Santa Cruz Biotechnology), and CSFV E2 (WH303; APHA) and polyclonal Abs recognizing ISG15 (sc-50366; Santa Cruz Biotechnology), Bax (2772; Cell Signaling Technology), cleaved caspase-3 (9664; Cell Signaling Technology), GFP (Ab290; Abcam), and FLAG (R1180; Acris) were all used as indicated. Bound primary antibodies were detected by horseradish peroxidase-conjugated goat anti-mouse (Promega) or goat anti-rabbit (Promega) secondary antibodies.

### Immunofluorescence.

Cells were prepared on coverglasses prior to treatments and fixed with 4 % paraformaldehyde (Santa Cruz Biotechnology) for 1 h, permeabilized with 0.1 % Triton X-100 for 5 min, and blocked in 10% goat serum (G9023; Sigma) in PBSa (lacking MgCl_2_ and CaCl_2_). As primary antibodies, a MAb recognizing CSFV E2 (WH303; APHA) and rabbit polyclonal Ab recognizing Bax (2772; Cell Signaling Technology) were used where indicated. Secondary antibodies were goat anti-mouse conjugated with Alexa Fluor 488 or 633 (Thermo Fisher). Nuclei were stained with DAPI (4′,6-diamidino-2-phenylindole; Sigma). For mitochondrial staining, 150 nM MitoTracker red CMXRos (Thermo Fisher) diluted in complete growth medium was added to the cells 30 min prior to washing in PBS and fixation. Prepared slides of cells were imaged on a Leica TCS SP2 Acousto-Optical beam splitter confocal scanning laser microscope at wavelengths appropriate for each Alexa Fluor probe.

Where specified, protein localization was quantified as follows: images were imported into ImageJ, and automated counting was used to determine the total number of nuclei per field of view. For Bax studies, cells demonstrating condensed mitochondrial localization were manually counted and divided by the count of nuclei to give the percentage of positives. Two-way analysis of variance (ANOVA; GraphPad) was used to determine mean, standard deviation (SD), and confidence interval (CI) for *n* = 5.

### Yeast two-hybrid analysis.

The Matchmaker 3 GAL4-based yeast two-hybrid system (Clontech Laboratories) was employed to identify direct protein-protein interactions. A cDNA encoding CSFV N^pro^ (Alfort) was cloned into the pGBKT7 and pGADT7 vectors to generate fusions with the GAL4 DNA-binding and activation domains, respectively. A cDNA encoding porcine IRF3 (GenBank accession no. NM_213770.1) was additionally cloned into pGADT7, while a cDNA encoding porcine Bax (GenBank accession no. XM_003127290.5), modified by PCR to lack the terminal 20 amino acids (Val^173^ to Gly^192^), was cloned into each vector. Yeast cells (AH109) were grown, maintained, and transformed as instructed by the manufacturer (Clontech Laboratories). Cotransformed yeast cultures were subsequently plated onto double-dropout medium deficient of leucine and tryptophan and quadruple-dropout medium additionally lacking adenine and histidine.
